# The Impact of Endurance Exercise on Routine Laboratory Parameters in Young Trained Individuals

**DOI:** 10.3390/jcm14165703

**Published:** 2025-08-12

**Authors:** Robert Nowak, Karolina Turkiewicz, Michał Sławiński, Jeremy S. C. Clark, Aleksandra Szylińska, Patrizia Proia, Łukasz Jodko, Bartosz Wojciuk, Violetta Sulżyc-Bielicka, Dorota Kostrzewa-Nowak

**Affiliations:** 1 Institute of Physical Culture Sciences, University of Szczecin, 17C Narutowicza St., 70-240 Szczecin, Poland; 2 Department of Pathology, Pomeranian Medical University in Szczecin, 1 Unii Lubelskiej St., 71-242 Szczecin, Poland; 3 Department of Clinical and Molecular Biochemistry, Pomeranian Medical University in Szczecin, 72 Powstańców Wlkp. Al., 70-111 Szczecin, Poland or karolina.turkiewicz@usk2.szczecin.pl (K.T.); or michal.slawinski@usk2.szczecin.pl (M.S.); jeremy.clark@pum.edu.pl (J.S.C.C.); lukasz.jodko@pum.edu.pl (Ł.J.); dorota.kostrzewa.nowak@pum.edu.pl (D.K.-N.); 4 Department of Laboratory Diagnostics, University Clinical Hospital No. 2, Pomeranian Medical University in Szczecin, 72 Powstańców Wlkp. Al., 70-111 Szczecin, Poland; 5 Department of Cardiac Surgery, Pomeranian Medical University, 72 Powstańców Wlkp. Al., 70-111 Szczecin, Poland; aleksandra.szylinska@pum.edu.pl; 6 Sport and Exercise Sciences Research Unit, Department of Psychology, Educational Science and Human Movement, University of Palermo, 90144 Palermo, Italy; patrizia.proia@unipa.it; 7 Department of Diagnostic Immunology, Chair of Microbiology, Immunology and Laboratory Medicine, Pomeranian Medical University in Szczecin, 72 Powstańców Wlkp. Al., 70-111 Szczecin, Poland; bartosz.wojciuk@pum.edu.pl; 8 Department of Clinical Oncology, Pomeranian Medical University, 4 Arkońska St., 71-455 Szczecin, Poland; violetta.sulzyc.bielicka@pum.edu.pl

**Keywords:** physical effort, laboratory medicine, blood morphology, clinical biochemistry, exercise biochemistry

## Abstract

**Background**: Endurance effort aims to improve aerobic capacity. During physical exertion, fluid shifts from intravascular to interstitial spaces, affecting potential conclusions from laboratory test results. The study aimed to assess the effects of endurance exercise on clinical interpretations of routine laboratory hematological and biochemical diagnostic tests. **Methods**: Participants were young, healthy, and physically active men aged 16–36 and women aged 16–29, who performed progressive treadmill tests to exhaustion. Blood samples were collected before the test, immediately after the test, and after 17 h of recovery. **Results**: The results showed that endurance exercise led to transient increases in the number of peripheral blood leukocytes and their subpopulations. A direct biological effect of endurance effort was an increase in the activity of amylase, AST, ALT, CK, GGT, LDH, and ALP, as well as in the concentration of creatinine, urea, uric acid, glucose, albumin, total protein, total cholesterol, HDL, triglycerides, sodium, chloride, phosphorus, and iron. Decreases in potassium and calcium (total and ionized) concentrations were also observed. **Conclusions**: The analyses clearly showed that laboratory tests performed in highly trained individuals may provide interpretation difficulties, and the reference ranges generally accepted in the healthy population might not apply to athletes.

## 1. Introduction

According to the Centers for Disease Control and Prevention (USA) and in the context of sports training, physical effort is a form of physical activity that is planned (usually by a professional) and repeated, the purpose of which is to improve and/or maintain physical fitness at a high level [1]. The World Health Organization has defined physical activity as a movement of the body produced by skeletal muscles. Therefore, this refers to any type of movement, not only including physical effort but also to everyday activities [2]. A physical exercise is a specific task performed to achieve physical activity or exertion.

As a result of a physical exercise, a number of functional changes occur in the body whose size and nature depend on, for example, the level of training, the type of exercise, its intensity, or its duration [3,4,5,6]. Repeated endurance training leads to a number of adaptive changes at the cellular level. These mainly concern an increase in the number of mitochondria, resulting in an increased ability to produce ATP through increased oxidative phosphorylation. At rest, the reserves of ATP in muscles are small [7,8,9], but an increase and subsequent continuous supply of ATP is needed during exercise [9,10], regardless of whether this is to last for seconds or hours (h).

Within 17 h after a physical exercise, there is an increase in the synthesis of alpha-aminolevulinic acid, considered to be the first symptom of enzymatic adaptation in response to physical exercise. With training, skeletal muscle hexokinase activity increases; the skeletal-muscle-specific isozyme of lactate dehydrogenase decreases, and the heart-muscle-specific isozyme increases in activity [11,12]. Muscle contractile activity during training increases muscle fiber plasticity and remodeling, allowing functional adaptation [10,13,14].

After endurance training, a general increase in mRNA expression in response to exercise facilitates the synthesis of some proteins, influencing muscle remodeling and adapting their structure to subsequent training loads [5]. For example, in human and rodent studies, moderate training causes a ~2× increase in HK II mRNA [12]. During molecular adaptive changes, there is a gradual increase in protein and enzymatic activity. This is caused by the activation or repression of specific signaling pathways regulating transcription and translation and the expression of exercise-responsive genes, such as the regulatory genes of carbohydrate and lipid metabolism and their transport and oxidation, mitochondrial metabolism, and oxidative phosphorylation [15,16,17]. The result of these adaptive changes is to optimize the body’s performance in terms of fatigue resistance while maintaining homeostasis in the face of metabolic disturbances [11,18,19].

One of the many adaptive processes to repeated endurance exercise is an increase in plasma volume, perhaps to hypervolemia, at rest (which will partially counter the loss in volume during exercise). This is related to the balance between hydrostatic and osmotic forces, i.e., passive water movement and active transport of osmotically active substances. The movement of water between the interstitial and intravascular spaces depends on this balance between hydrostatic and oncotic pressure, with the latter mostly dependent on the plasma albumin concentration. Repeated endurance exercise increases plasma volume, often by 8–12%, mainly through plasma, with delayed red-cell mass gains. Due to the ability of this protein to affect osmotic pressure, changes in the albumin distribution between body compartments play a key role in changing plasma volume in training individuals [20,21,22]. Water and salt retention after exercise is also affected by increases in the activities of plasma renin and vasopressin [23].

Exercise increases capillary hydrostatic pressure and intramuscular osmolality (from metabolites like lactate, K^+^, and phosphate), leading to net filtration of plasma into the interstitial space. Elevated cardiac output also increases capillary permeability to proteins, promoting outward fluid shifts and resulting in hemoconcentration (~1 g hemoglobin per 100 mL). During physical exercise, the intramuscular metabolite content and extravascular osmolality increase, which, together with an increase in blood pressure and activation of the sympathetic nervous system, cause a fluid shift from the vascular space to the interstitial space, in proportion to protein and electrolyte concentrations [23,24,25,26]. This causes a decrease in plasma volume and the phenomenon of hemoconcentration [27,28]. During prolonged exercise of increasing intensity, both the metabolic rate and heat production increase, which also reduces the plasma volume during physical exercise [26,29]. Increasing the intensity of an exercise results in an increased loss of plasma volume [28,30].

Changes in blood biomarker concentrations caused by physical exercise can result from tissue stresses in response to physical activity and/or from hemoconcentration. Loss of plasma volume causes an increase in the concentration of circulating biomarkers regardless of their response to tissue stresses [30,31]. Therefore, it is essential to define the post-exercise loss of plasma volume to determine an adjusted effect of exercise on changes in the concentrations or activities of circulating biomarkers [32].

Response to physical exercise provides one component of biological variability, i.e., the physical changes can depend on the individual subjects. This biological variability depends on parameters such as sex, age, diet, stress, body characteristics, circadian rhythm, and lifestyle [33]. For this reason, laboratory test results in physically active people should be interpreted with great caution, and extrapolation to the general population might not be possible because results may deviate from accepted reference ranges as a result of these subjects’ adaptation to regular physical exercise or as a direct consequence of exercise. Similarly, changes might not apply to pathology, injury, or disease [32].

The body’s response to physical effort is related to the level of training. The same series of exercises will cause a different scale of changes in biochemical or hematological parameters in a fit athlete than in a person who leads a sedentary lifestyle [34]. Thus, in an untrained person, after a specific training load, an apparent change in the results of biomarker analysis might be visible. In contrast, in a person who regularly exercises, this might only be visible as a result of more intensive physical effort [35]. The present study has analyzed parameters from a group of young, healthy, trained people. These individuals, although not athletes, had been trained to a level at which the exercises they could perform could be dangerous to apply to people with a normally sedentary lifestyle.

From the above, the reference ranges from a healthy population leading a sedentary lifestyle are difficult to translate to highly trained individuals or recreational athletes, and their application to competitive athletes may even be misleading [36]. However, knowledge about the dynamics of changes in biomarkers in the blood of highly trained people might allow for generalization to other highly trained individuals or athletes, preventing unnecessary additional tests and limiting the requirement for study tests during training and sports competitions. Additionally, this knowledge might be of use for clinicians who encounter such subjects. Information from the patient about the time that has passed since the last training before blood sampling and the frequency of training is necessary when interpreting laboratory test results to avoid misdiagnosis.

Hence, this study aimed to assess the effects of endurance exercise on the clinical interpretation of routine laboratory hematological and biochemical diagnostic tests and especially (i) to try to distinguish the effects of hemoconcentration from tissue effects, especially in young men, and (ii) to provide further data to clinicians, including some data from women. The parameters included the following: metabolite concentrations: glucose, urea, creatinine, uric acid, and bilirubin (total and direct); albumin, total protein, and C-reactive protein; the lipid profile: triglycerides and cholesterol: total, high-density, and low-density lipoprotein concentrations; enzyme activities: aspartate and alanine aminotransferases, gamma-glutamyltransferase, alkaline phosphatase, creatine kinase, lactate dehydrogenase, and amylase; and selected ions.

## 2. Materials and Methods

### 2.1. Participants

In this study, 296 participants (234 men and 62 women) were recruited from team sports, from football and handball sports clubs. The participants included trained, young, healthy subjects of European descent. The exclusion criteria were metabolic syndrome, cardiovascular diseases, immunodeficiencies, and endocrinological-related diseases. Additionally, all participants were non-smokers and refrained from taking any medications or supplements known to affect metabolism. Physical fitness level was designated based on a participant’s declaration of weekly training volumes and training experience. The participants were familiar with the protocols of the exercises to be performed, and they took part in the experiment after two days of recovery from the effort. They (and their parents if appropriate) were fully informed of any risks and discomfort associated with the experimental procedures before giving their written consent to participate. The study was approved by the local Ethics Committee at the Regional Medical Chamber in Szczecin (approval no. 05/KB/VII2019) and followed the latest Declaration of Helsinki (2024).

### 2.2. Exercise Tests and Blood Sampling

All participants performed the same progressive efficiency test on a mechanical treadmill until exhaustion. The test was performed in the morning under laboratory conditions, two hours after a light breakfast and after two days of recovery, at a temperature of 20–23 °C. The proper test was preceded by a five-minute (min) warm-up run. During the proper test, the speed started at 5 km/h and was increased by 2 km/h every 3 min (min) of the test until exhaustion, meaning that the subject was unable to continue the run and had achieved that individual’s maximum fatigue. Further details have been previously described [37,38].

Blood samples were obtained three times from an elbow vein: 5 min before the exercise test (pre), no longer than 5 min after the exercise test (post), and about 17 h after the test, at the end of the recovery period (rec). It should be noted that, for safety reasons, the test protocol required the participant to have a light breakfast. Therefore, blood samples collected before (pre) and after (post) the test were not fasting blood, whereas the third (fasting) blood sample (rec) was drawn in the morning before breakfast. Each time, blood samples were drawn into two tubes: for serum preparation, a 7.5 mL S-Monovette tube with a clot activator (SARSTEDT AG & Co., Nümbrecht, Germany) was used, and for complete blood count and plasma preparation, a 9 mL S-Monovette tube with ethylenediaminetetraacetic acid (EDTA K3, 1.6 mg EDTA/mL blood; SARSTEDT) was used. Blood samples to obtain serum or plasma were centrifuged at 2000× *g* for 10 min at room temperature. Biochemical and hematological analyses were performed immediately after blood collection.

### 2.3. Medical Laboratory Methods

#### 2.3.1. Hematological Analyses

Complete blood counts, including assessments of amounts of white blood cells (WBC), red blood cells (RBC), hemoglobin (HGB), hematocrit (HCT), mean corpuscular volume (MCV), mean corpuscular hemoglobin (MCH), mean corpuscular hemoglobin concentration (MCHC), and total platelet levels (PLT), were obtained using a hematology analyzer ABX Micros 60 (Horiba ABX, Warsaw, Poland).

#### 2.3.2. Biochemical Analyses

Biochemical analyses were conducted for clinical chemistry variables (using an Auto Chemistry Analyser BM-100; BioMaxima, Lublin, Poland) or for ions (using an Ion Selective Analyser BM ISE; BioMaxima). From blood plasma, the following parameters were determined: metabolite concentrations of creatinine, uric acid, and bilirubin (total and direct); albumin, total protein, and C-reactive protein (CRP); the lipid profile: triglycerides (TG), cholesterol: total (TC), high-density (HDL-C or Ch-HDL), and low-density lipoprotein (LDL-C or Ch-LDL) concentrations; enzyme activities: aspartate (AST) and alanine (ALT) aminotransferases, gamma-glutamyltransferase (GGTP), alkaline phosphatase (ALP), creatine kinase (CK), lactate dehydrogenase (LDH), and amylase. The serum was used for determining the concentrations of glucose, urea, and ions.

All the studied variables were determined using diagnostic methods according to the appropriate manufacturer’s protocols (BioMaxima). All analyses were compared with a multiparametric panel of control sera, including control sera with normal levels (BioNorm) and with high levels (BioPath) (both from BioMaxima).

### 2.4. Calculations and Statistical Analyses

Sera osmolalities were calculated using the mmol/L concentrations of Na^+^, glucose, and urea according to the following formula [39,40]:

$$\mathrm{Total}\;\mathrm{osmolality}\;\left(\mathrm{mOsm}/L\right)=2\times \left[{Na}^{+}\right]+\left[glucose\right]+\left[urea\right]$$

Moreover, to compensate for changes in analyzed variables due to hemoconcentration, plasma volume loss (ΔPV) was calculated according to the equation from Dill and Costill, provided by Alis et al. [27], as follows:

$$∆PV\left(\%\right)=100\times \left(\frac{{Hb}_{pre}}{{Hb}_{post}}\times \frac{100-{Htc}_{post}}{100-{Htc}_{pre}}-1\right)$$

where Hb_pre_ = hemoglobin pre-test (g/dL); Hb_post_ = hemoglobin post-test (or in recovery) (g/dL); Htc_pre_ = hematocrit pre-test (%); and Htc_post_ = hematocrit post-test (or in recovery) (%).

The formula for the correction of blood parameters was as follows [27]:

$$\left[Corrected\;parameter\;concentration\right]=\left[Uncorrected\;parameter\;concentration\right]\times \left(1+\frac{∆PV(\%)}{100}\right)$$

Statistical analyses were performed using Statistica (version 13, 2017; TIBCO, Palo Alto, CA, USA). The normality of the data distribution was assessed using the Shapiro–Wilk test. Since the data did not follow a normal distribution, non-parametric tests were used, and all data are presented as medians (interquartile range) except for age, which is presented as the median (minimum–maximum range). Changes between analyzed time points (baseline vs. post-effort vs. recovery) were assessed using Friedman’s analysis of variance for repeated measures, followed by post-hoc Dunn’s tests with Bonferroni correction.

## 3. Results

**Figure 1:**
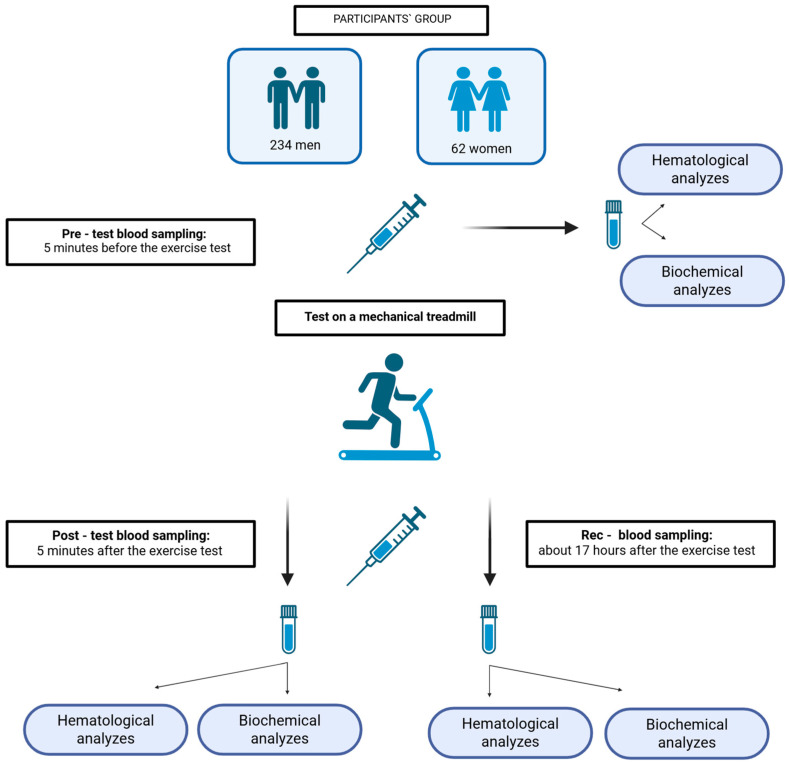
Study design model (Created in BioRender; https://BioRender.com/7ezeeal; accessed on 26 June 2025).

To solve the scientific problem, young, trained, and healthy volunteers were recruited and performed the endurance effort on a mechanical treadmill until exhaustion. The blood samples for all analyzes were collected at three time points according to the study protocol presented in Figure 1.

The general characteristics by sex of the participants are presented in Table 1. The most essential data are those for men (due to the larger number of participants). The data for women are provided mainly for comparison. There were no unexpected differences between the sexes.

For the convenience of the reader, all the datasets discussed in this paper are presented in Supplementary Table S1.

With baseline blood morphology (Table 2), the significant differences observed between men and women were similar to those commonly found due to physiological sex differences (Table 2). In both groups, significant post-effort increases in RBC, HTC, and HGB were found, while after 17 h of recovery, these parameters were found to be significantly lower than baseline values. These results are directly related to a plasma volume decrease (ΔPV)—a hypovolemic effect found with this type of physical effort observed in both studied groups. Moreover, as a direct post-effort effect, the endurance effort caused leucocytosis (WBC) with lymphocytosis (LYM) and granulocytosis (GRA) in both groups. Only in the male group was monocytosis (MON) found as a post-effort effect in terms of both percentages and counts. It is worth noting that, after recovery time, the WBC and GRA counts were significantly lower at the recovery time point only in the female group, while in the male group, these values were similar to baseline. The fluctuation in platelet parameters showed that, directly after the endurance effort, the platelet count (PLT), mean platelet volume (MPV), and plateletcrit (PCT) were significantly higher than baseline values in both sexes. Significantly slightly lower values for these parameters were found as post-recovery observations in both groups (Table 2).

**Figure 2:**
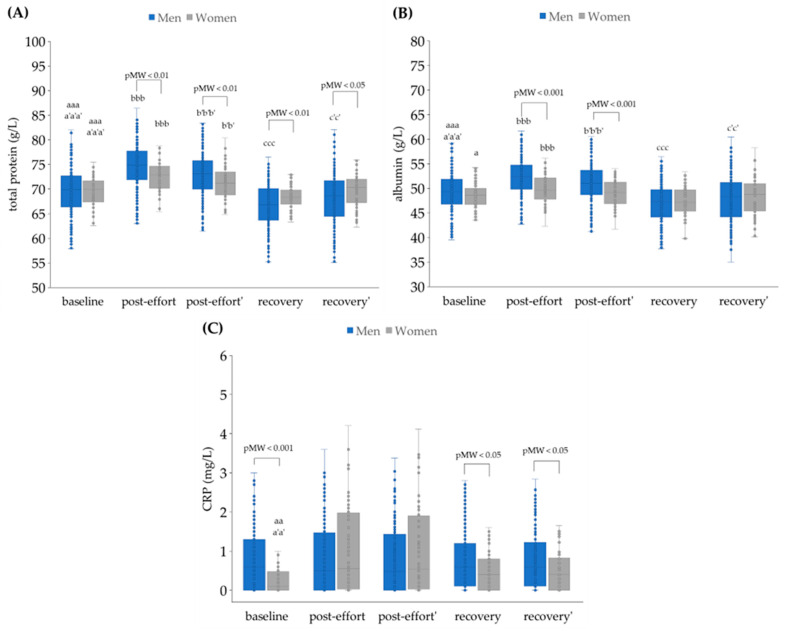
Plasma concentrations of (**A**) total protein, (**B**) albumin, and (**C**) C-reactive protein (CRP) in analyzed participants. Post effort’, recovery’ - values corrected for hemoconcentration. pMW: *p*-values for differences observed between men and women assessed using the Mann–Whitney U-test. Significance levels of differences observed between analyzed time points (baseline vs. post-effort vs. recovery) were assessed using Friedman’s analysis of variance, followed by post-hoc Dunn’s test with Bonferroni correction (Friedman’s ANOVA *p* values for each analysis were <0.001). Post-hoc *p* values: ^a^ *p* < 0.05, ^aa^ *p* < 0.01, and ^aaa^ *p* < 0.001 for baseline vs. post-effort; ^bbb^ *p* < 0.001 for post-effort vs. recovery; ^ccc^ *p* < 0.001 for recovery vs. baseline; ^a’a’^ *p* < 0.01, and ^a’a’a’^ *p* < 0.001 for baseline vs. post-effort values corrected for hemoconcentration; ^b’b’^ *p* < 0.01, and ^b’b’b’^ *p* < 0.001 for post-effort vs. recovery values corrected for hemoconcentration; ^c’c’^ *p* < 0.01 for post-effort vs. recovery values corrected for hemoconcentration.

It was found that the endurance effort caused a significant increase in total protein (TP) and albumin concentration in both sexes, which was not only related to the loss of plasma volume (Figure 2A). After recovery time, both TP and albumin decreased significantly in the men, which was only partially corrected for by hemoconcentration. The data for women did not show this statistically significant fall for total protein. In general, the concentrations of TP and albumin were significantly lower in women’s plasma in comparison to men’s at all studied time points (Figure 2A,B). The post-effort and recovery CRP concentrations did not differ across time points when the effect of plasma volume loss was considered (Figure 2C).

**Figure 3:**
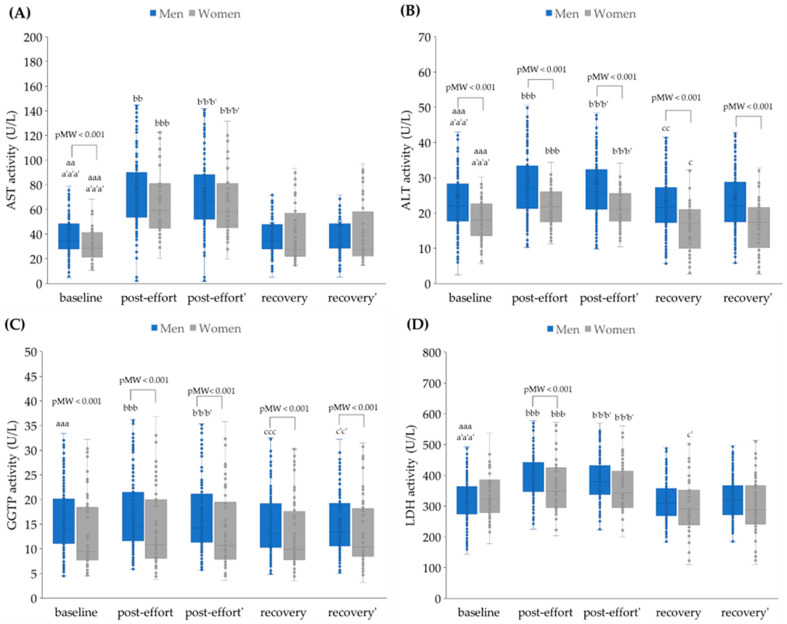

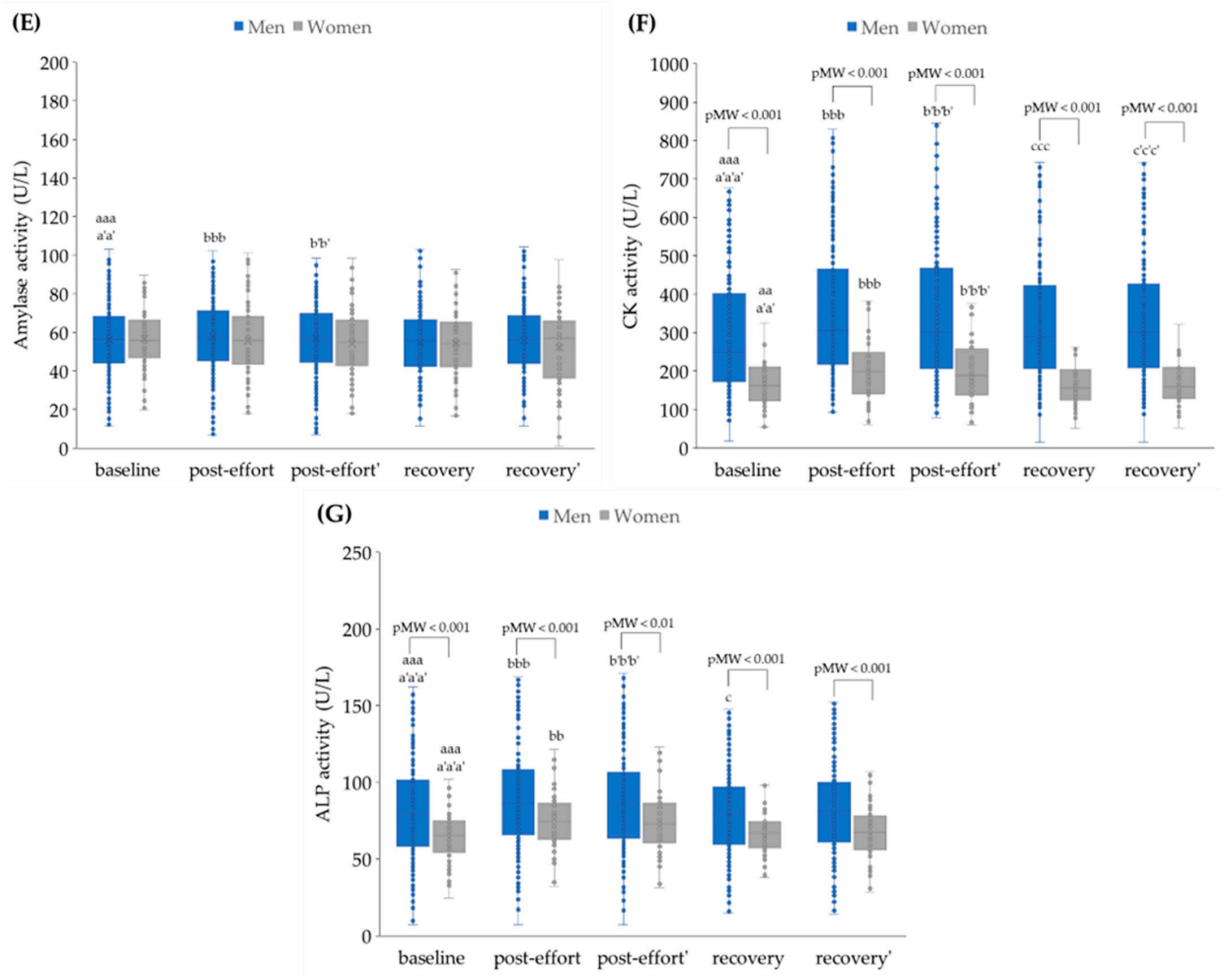
The plasma activity of (**A**) aspartate aminotransferase (AST), (**B**) alanine aminotransferase (ALT), (**C**) gamma-glutamyltransferase (GGTP), (**D**) lactate dehydrogenase (LDH), (**E**) amylase, (**F**) creatine kinase (CK), and (**G**) alkaline phosphatase (ALP) in analyzed participants. Post effort’, recovery’ - values corrected for hemoconcentration. pMW: *p*-values for differences observed between men and women assessed using the Mann–Whitney U-test. Significance levels of differences observed between analyzed time points (baseline vs. post-effort vs. recovery) were assessed using Friedman’s analysis of variance, followed by post-hoc Dunn’s test with Bonferroni correction (Friedman’s ANOVA *p* values for each analysis were <0.001). Post-hoc *p* values: ^aa^ *p* < 0.01 and ^aaa^ *p* < 0.001 for baseline vs. post-effort; ^bb^ *p* < 0.01 and ^bbb^ *p* < 0.001 for post-effort vs. recovery; ^c^ *p* < 0.05, ^cc^ *p* < 0.01, and ^ccc^ *p* < 0.001 for recovery vs. baseline; ^a’a’^ *p* < 0.01, and ^a’a’a’^ *p* < 0.001 for baseline vs. post-effort values corrected for hemoconcentration; ^b’b’^ *p* < 0.01, and ^b’b’b’^ *p* < 0.001 for post-effort vs. recovery values corrected for hemoconcentration; ^c’^ *p* < 0.05, ^c’c’^ *p* < 0.01, and ^c’c’c’^ *p* < 0.001 for post-effort vs. recovery values corrected for hemoconcentration.

Endurance effort caused a significant increase in AST, ALT, and ALP activities in both sexes’ plasma, and this effect was not related to post-effort plasma volume loss. After 17 h of recovery, the activities of these enzymes were similar to baseline values (Figure 3A,B,G). The significant changes in GGTP and amylase activities were found only in the male group (Figure 3C,E). The post-effort (both non-corrected and corrected) activity of LDH was significantly higher in both groups of participants. However, recovery activities were significantly lower in comparison to baseline ones only in the women’s group (Figure 3D). The CK activity was higher than baseline values in all studied time points in the men’s group and only in the post-effort time point (both non-corrected and corrected) among the women’s group. Moreover, in men, higher values with greater scatter were observed for CK activity at each studied time point, compared to women (Figure 3F).

**Figure 4:**
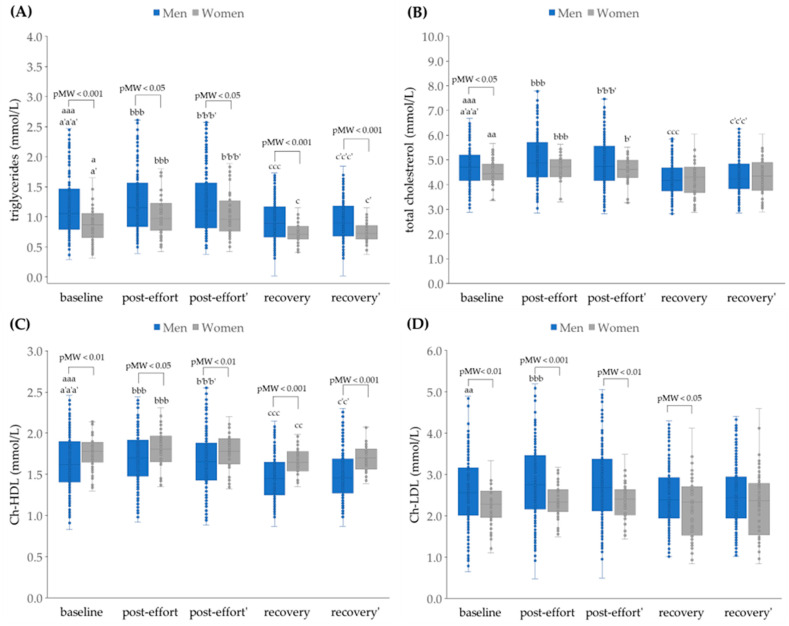
The plasma concentrations of (**A**) triglyceride (TG), (**B**) total cholesterol (TC), (**C**) high-density lipoprotein cholesterol (Ch-HDL), and (**D**) low-density lipoprotein (Ch-LDL) in analyzed participants. Post effort’, recovery’ - values corrected for hemoconcentration. pMW: *p*-values for differences observed between men and women assessed using the Mann–Whitney U-test. Significance levels of differences observed between analyzed time points (baseline vs. post-effort vs. recovery) were assessed using Friedman’s analysis of variance, followed by post-hoc Dunn’s test with Bonferroni correction (Friedman’s ANOVA *p* values for each analysis were <0.001). Post-hoc *p* values: ^a^ *p* < 0.05, ^aa^ *p* < 0.01, and ^aaa^ *p* < 0.001 for baseline vs. post-effort; ^bbb^ *p* < 0.001 for post-effort vs. recovery; ^c^ *p* < 0.05, ^cc^ *p* < 0.01, and ^ccc^ *p* < 0.001 for recovery vs. baseline; ^a’^ *p* < 0.05, and ^a’a’a’^ *p* < 0.001 for baseline vs. post-effort values corrected for hemoconcentration; ^b’^ *p* < 0.05, and ^b’b’b’^ *p* < 0.001 for post-effort vs. recovery values corrected for hemoconcentration; ^c’^ *p* < 0.05, ^c’c’^ *p* < 0.01, and ^c’c’c’^ *p* < 0.001 for post-effort vs. recovery values corrected for hemoconcentration.

**Figure 5:**
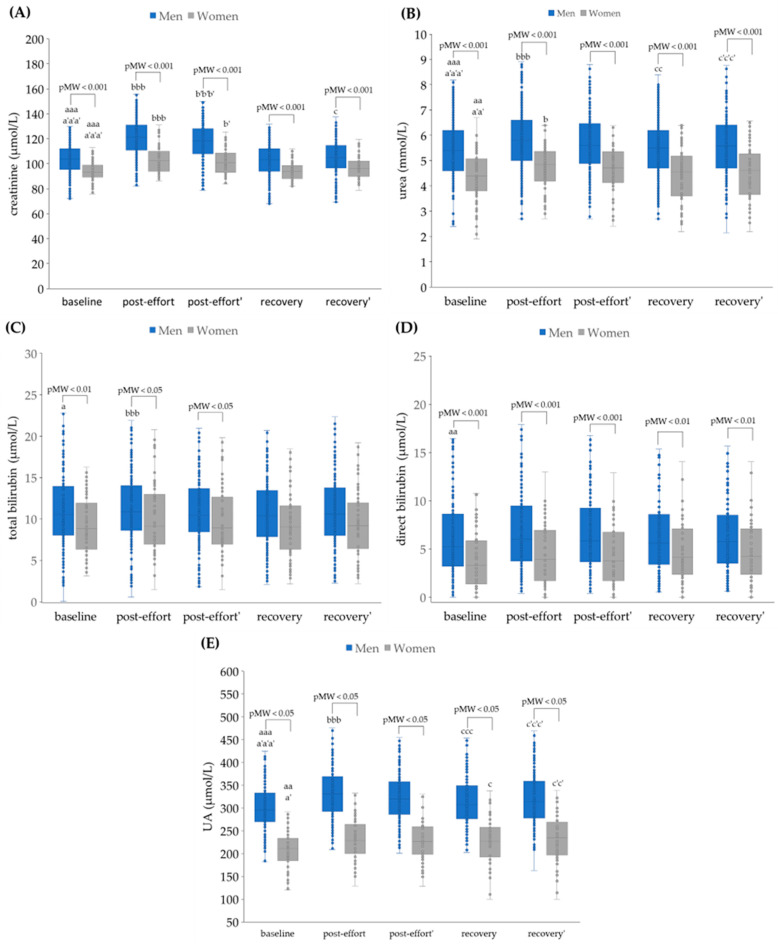
Plasma concentrations of (**A**) creatinine, (**B**) urea, (**C**) total bilirubin, (**D**) direct bilirubin, and (**E**) uric acid (UA) in analyzed participants. Post effort’, recovery’—values corrected for hemoconcentration. pMW: *p*-values for differences observed between men and women assessed using the Mann–Whitney U-test. Significance levels of differences observed between analyzed time points (baseline vs. post-effort vs. recovery) were assessed using Friedman’s analysis of variance, followed by post-hoc Dunn’s test with Bonferroni correction (Friedman’s ANOVA *p* values for each analysis were <0.001). Post-hoc *p* values: ^a^ *p* < 0.05, ^aa^ *p* < 0.01, and ^aaa^ *p* < 0.001 for baseline vs. post-effort; ^b^ *p* < 0.05 and ^bbb^ *p* < 0.001 for post-effort vs. recovery; ^c^ *p* < 0.05, ^cc^ *p* < 0.01, and ^ccc^ *p* < 0.001 for recovery vs. baseline; ^a’^ *p* < 0.05, ^a’a’^ *p* < 0.01, and ^a’a’a’^ *p* < 0.001 for baseline vs. post-effort values corrected for hemoconcentration; ^b’^ *p* < 0.05, and ^b’b’b’^ *p* < 0.001 for post-effort vs. recovery values corrected for hemoconcentration; ^c’c’^ *p* < 0.01, and ^c’c’c’^ *p* < 0.001 for post-effort vs. recovery values corrected for hemoconcentration.

Generally, the progressive effort caused statistically significant changes in lipid profile (apart from Ch-LDL) in men (Figure 4) with increases from baseline to post-effort and decreases from post-effort (and baseline) to recovery. Some statistical differences, e.g., between baseline and recovery for total cholesterol, were not seen in the group of women, possibly because of the lower numbers. Ch-LDL and creatinine concentrations (Figure 5A) did increase between baseline and post-effort and then decrease again but at recovery were not significantly different from baseline.

Urea and uric acid concentrations (Figure 5) significantly increased post-effort with uncorrected values, but after hemocorrection, no statistically significant difference was found between baseline and post-effort. However, after 17 h of recovery, urea and uric acid concentrations had increased significantly from baseline in men (the data for women did not show statistical significance), even when corrected for hemoconcentration.

No statistically significant changes after correction for hemoconcentration were found with total and direct bilirubin concentrations (Figure 5).

It was found that endurance effort caused significant increases in Na^+^, K^+^, and Cl^−^ concentrations in both sexes (Table 3). However, the initial effects (post-effort) were found to be only related to the impact of loss of plasma volume because, after correcting post-effort values according to the equations from Dill and Costill, opposing effects were observed. However, it was found that after 17 h of recovery, the values (both corrected and non-corrected) were significantly higher than baseline values (Table 3).

A significant decrease in calcium concentrations (total and free unbound ionized) was found as a post-effort effect in both sexes, regardless of plasma volume loss. After recovery, the calcium concentrations moved in the opposite direction, i.e., were significantly higher than baseline values, in both men and women (Table 3).

**Figure 6:**
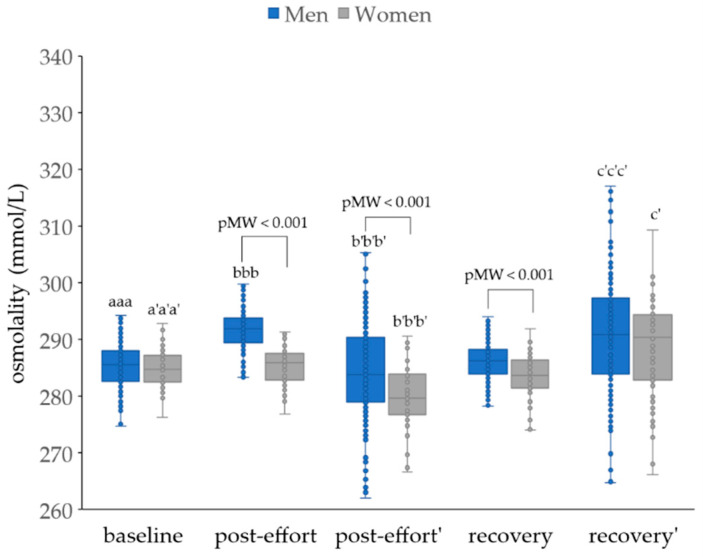
Calculated osmolalities in analyzed participants. Post effort’, recovery’ - values corrected for hemoconcentration. pMW: *p*-values for differences observed between men and women assessed using the Mann–Whitney U-test. Significance levels of differences observed between analyzed time points (baseline vs. post-effort vs. recovery) were assessed using Friedman’s analysis of variance, followed by post-hoc Dunn’s test with Bonferroni correction (Friedman’s ANOVA *p* values for each analysis were <0.001). Post-hoc *p* values: ^aaa^ *p* < 0.001 for baseline vs. post-effort; ^bbb^ *p* < 0.001 for post-effort vs. recovery; ^a’a’a’^ *p* < 0.001 for baseline vs. post-effort values corrected for hemoconcentration; ^b’b’b’^ *p* < 0.001 for post-effort vs. recovery values corrected for hemoconcentration; ^c’^ *p* < 0.05, and ^c’c’c’^ *p* < 0.001 for post-effort vs. recovery values corrected for hemoconcentration.

Calculated osmolalities were significantly higher in the men group in all studied time points, while in the women group, they were significantly lower after endurance effort. With corrected values, these parameters were higher than baseline values after 17 h of recovery (Figure 6).

## 4. Discussion

### 4.1. The Impact of Endurance Effort on Blood Morphology

Physical effort causes an increase in several red blood cell parameters, including the red blood cell count, as a result of hemoconcentration [41,42]. In the present study, it was observed that an endurance test on a mechanical treadmill until exhaustion caused a transient increase in the RBC count in both sexes, as found in a previous study with an endurance test on a rowing ergometer [43]—a transient increase in the RBC count and a return to baseline values 24 h after the test. Similar results have also been found in basketball players subjected to a running test on a mechanical treadmill [44]. An increase in the RBC count was observed after exercise, but after three hours, the values were lower than baseline values [44]. Different results were observed in a study of athletes who underwent extremely high physical effort (completion of a 24-h ultramarathon)—RBC counts after the race did not show statistically significant deviations compared to a pre-exercise study but did decrease significantly on the second and ninth day after the race, reaching the lowest value on the second day of observations [45]. Another study examining the marathon effect also showed a significant decrease in RBC counts, occurring four hours after the race and lasting up to 24 h [41]. This phenomenon could be caused by accelerated destruction of red blood cells (intravascular hemolysis), which is a common effect in endurance athletes subjected to such intense physical effort [45,46].

Intense effort usually triggers leukocytosis [47,48]. Increases in white blood cell counts are associated with increases in subpopulations, as was the case in the present study. Similar results have been reported in a study of marathon runners, where an increase in WBC count was noted 4 h after the race and a decrease after 24 h [41]. Running a 24-h ultramarathon also increased WBC counts immediately after the race, but these values were maintained until the 9th day after its completion [45]. In a study of amateur runners who completed a 5-h run, an increase in WBC count was noted within an hour of its completion [47]. The increase in WBC counts is most likely associated with an increase in the neutrophil population [32]. Granulocyte counts, as in the present study, increased after the 24-h ultramarathon (400 km) and decreased to baseline values on the second day after the race [45]. The same relationship was observed after the marathon, where an increase in the number of granulocytes was noted after 4 h and a decrease after 24 h after finishing the marathon [41].

Lymphocyte counts decreased immediately after the 24-h ultramarathon but returned to normal on the second day [45]. A similar effect was obtained in marathon participants—a decrease after 4 h and an increase after 24 h [41]. The results of these studies were different from those in men in our studies, in which the number of lymphocytes increased after the endurance test and decreased to baseline values after 17 h of recovery.

Monocyte counts have been found to increase, as in the present study, after running a 24-h ultramarathon but only returned to normal on the 9th day after finishing the race [45].

A similar effect was observed in the study of a marathon, where the number of monocytes increased after 4 h and remained for up to 24 h after finishing the race [41].

A 50-min intensive endurance exercise on a rowing ergometer led to an increase in the WBC count compared to baseline values, immediately after and 3 h after the test. This was associated with an increase in neutrophil and monocyte populations, while lymphocyte counts increased after the test and dropped to baseline values three hours after its completion [49]. In the present study, a transient increase in the platelet count was noted. A study analyzing marathon runners showed an almost identical effect of exercise on the PLT count—an increase after 4 h and a decrease after 24 h after the end of the marathon [41]. In a study of participants in a 24-h ultramarathon, the PLT count increased immediately after the race and on the second day dropped below baseline values [45].

All noted sex differences were comparable with well-described physiological sex differences.

### 4.2. The Impact of Physical Effort on Selected Biochemical Variables

#### 4.2.1. Plasma Volume Changes as a Post-Effort Factor

It has been shown that an increase in plasma volume within 24 h after intensive interval exercise is caused by an increase in the concentration of albumin in the plasma, which results in the movement of water from the interstitial space to the vascular space [20,50,51]. Studies have shown that as a result of intensive exercise, albumin moves to the vascular bed from stores accumulated in the interstitial space through lymphatic vessels. At the same time, the escape of this protein through the capillaries is hindered [52,53]. The results of the present study showed that in both sexes, the concentration of albumin in the plasma increased after exercise. After a 17-h recovery, it dropped to a level lower than before the test.

These results are similar to a previously conducted test of constant intensity on a mechanical treadmill, where an increase in albumin concentration immediately after exercise and a decrease to baseline values 30 min after the test were found [31]. Additionally, correction of our presented results for the change in plasma volume showed that corrected differences were insignificant [31], which is in line with previous studies. Studies of marathon participants showed significant increases in albumin concentrations 4 h after the race and a decrease to baseline values at 24 h [41]. Moreover, a 24-h ultramarathon did not show significant changes in albumin levels immediately after the race compared to the values before the race. On the 2nd and 9th days after the marathon, albumin concentrations showed a significant decrease [45]. Among trained marathon runners, after running a two-day marathon, the results showed significant increases in albumin concentrations on the first and second day after the race and decreases to the baseline values noted only on the third day [54]. In a comparative analysis of a marathon, a 100 km, and a 302 km run, significant increases in albumin concentrations were observed in people after running a marathon compared with the other races. Interestingly, a decrease in albumin concentrations was observed in runners who covered 302 km [55]. An acute renal response to high-intensity physical exercise seems to complement the effect of albumin on increasing plasma volume [56].

The hemoglobin and hematocrit measurements provide an easy way to check the degree of changes in plasma volume caused by various clinical conditions and in those caused by physical exercise [32]. In the present study, hemoglobin concentrations and hematocrit values in both groups changed significantly—they increased immediately after exercise and decreased after a 17-h restitution period. An increase in hemoglobin concentrations has previously been observed in marathon runners 4 h after running a marathon and a decrease after 24 h. At the same time, a decrease in hematocrit values was demonstrated at these time intervals [41]. Similar conclusions were drawn in a study of professional basketball players subjected to progressive endurance exercise on a treadmill, showing an increase in hemoglobin concentrations and hematocrit values after the test and a decrease after 3 h compared to baseline values [44]. In 2015, a group of athletes were given an endurance test consisting of rowing 2000 m on a rowing ergometer. Increases in HGB concentrations and HCT values were observed immediately after exercise, with decreases to baseline values after 24 h of restitution [43]. Different results were obtained in participants of a 24-h ultramarathon who ran 400 km. No changes in HCT and HGB values were observed immediately after the race, with decreases compared to baseline values observed on the second day after the race [45]. Also, in a study of participants in a 2-day ultramarathon, it was shown that on the first and second day after the race, neither HGB concentrations nor HCT values changed significantly [54].

#### 4.2.2. The Changes in Activities of Selected Enzymes as a Post-Effort Effect

Physical effort contributes to many changes in various body systems, often translating into the release of enzymes, metabolic products, or ions into the bloodstream [32,57,58]. Changes in fluid volume can affect the concentration of substances dissolved in the plasma, causing an increase in concentration or dilution [59].

In recent years, many studies have been conducted on the effects of various sports disciplines on basic biochemical parameters. These analyses used different protocols and, for example, blood was collected for testing at different time intervals after the end of physical activity. Many published articles have made various conclusions regarding changes in the concentrations of biochemical parameters and enzyme activity resulting from exercise and the restitution period. In many analyses, the study groups have been differentiated in terms of age and sex, or they were characterized by different levels of training and endurance.

It is known that exercise and physical training cause microdamage to skeletal muscles [35,60,61]. In this study, the activities of enzymes that are associated with muscle damage resulting from increased physical activity in athletes, i.e., CK, AST, and LDH, were examined. For both sexes, significant changes resulting from the effort and the recovery period were observed.

The most widely described enzymatic change in sports diagnostics is the change in CK activity, the activity of which increases proportionally to the intensity and duration of exercise [60,61]. In the present study, CK activity increased after exercise and decreased after restitution but remained at a higher level than before the test. Similar results have been previously obtained in people subjected to intensive and long-term physical exercise, such as a marathon. A gradual increase in CK activity has been observed, followed by a decrease at 4 and 24 h post-run [41]. In a study of participants after a 2-day marathon, CK activity increased until the second day, then gradually decreased until the seventh day, when it reached a value close to the initial value [54]. Shin et al., in their comparison of long-distance runners, showed that the degree of increase in CK activity depends on the distance, i.e., on the intensity of the exercise [55]. However, shorter exercise and lower intensity also result in an increase in the activity of this enzyme in blood serum. In a study of 24 healthy men who underwent an endurance test to exhaustion on a treadmill, an increase in CK activity was found from the initial value immediately and 30 min after the exercise [27]. In a study of 20 athletes who underwent a test on a rowing ergometer, a gradual increase in CK activity was found immediately and 1 day after the test [43]. Different results were obtained after testing football players after playing a football match—no increase in CK activity was noted. It is worth noting, however, that the initial activity of this enzyme in the football players exceeded the accepted laboratory reference ranges in the healthy population [62]. A group of other football players who underwent a 60-min running test also did not notice any changes in CK activity after exercise and restitution [63].

AST activity in the general population is associated with disorders of liver metabolism. In athletes, this parameter helps assess the work and fatigue of muscles undergoing physical exercise [32,63]. In our studies, AST activity in both groups increased after the endurance test and decreased after 17 h to values close to the initial values. Similar results were obtained in participants of a 24-h ultramarathon, where AST activity increased immediately after the race, decreased after 2 days, and returned to normal on the 9th day [45]. One previous study has demonstrated an increase in AST activity the day after a match in young football players [62]. Running a half-marathon by men not professionally involved in sports also resulted in an increase in AST activity immediately after the race and at 3 h, 6 h, and 24 h [64]. In marathon runners, an increase in activity was demonstrated after 4 h and 24 h [41]. In participants of a 2-day marathon, an increase in AST activity was observed only on the second and third day after the race, and a decrease was observed on the fifth day, returning to the baseline values on the seventh day [54]. Shin et al., in 2016, showed a significant increase in AST activity in participants of a marathon, 100 km run, and 302 km run immediately after the race, noting that the degree of increase was related to the physical load on the participants [55]. The increase in the activity of this enzyme is most likely due to its increased secretion from damaged skeletal muscles. Similarly to AST, lactate dehydrogenase can be associated with liver disorders in the general population, and in athletes, its activity increases as a result of physical activity [58]. LDH activity in men studied showed an increase as a result of the exercise test and a return to the baseline values associated with restitution. Similar results were achieved in a study of the effect of running on a mechanical treadmill to fatigue, where an increase in LDH activity was observed after the test and a decrease 30 min after its completion [16]. In football players who performed a 60-min run in the open air, an increase in LDH activity was observed after the run and a decrease to the baseline values after restitution [65]. Also, football players who were tested after playing a match achieved an increase in LDH activity [62]. In professional female basketball players subjected to exercise to full fatigue on a mechanical treadmill, an increase in LDH activity was observed after exercise and a decrease to the baseline values 3 h after its completion [44]. Interestingly, after a 24-h marathon, LDH activity in participants was observed to increase after the race and only began to decrease on days 2 and 9 after its completion, remaining higher than the activity measured before the race [45]. After a two-day marathon, LDH activity increased on days 1 and 2 and decreased on days 3 to 7 but did not reach pre-race values [54]. Similarly, in a study of long-distance running, it was proven that the length and intensity of exercise affect the degree of increase not only in CK activity but also in LDH [55].

Assessing changes in the health condition of athletes based on laboratory tests can be difficult. In the present study, we decided to examine the effect of physical exercise on the activity of amylase, an enzyme associated with the functioning of the pancreas. In both sexes, an increase was observed after exercise and a decrease to baseline values after restitution. However, after correcting for the correction factor, these results, expressed as %ΔPV, showed that these changes were due to hemoconcentration rather than tissue production effects.

Studies on the effect of physical exercise on amylase activity are quite limited, as it is challenging to interpret changes in its activity with exercise. A study from 2020 analyzed the effect of an 8-week training plan, consisting of various training modules, on the activity of enzymes, including amylase. It was then shown that the activity of this enzyme varied after each training module and remained higher than the initial activity [66].

The analysis of the effect of physical effort on liver function in our studies consisted of the examination of ALT and GGTP activities and the concentrations of direct and total bilirubin. However, the latter may also increase in cases of excessive destruction of erythrocytes and release of hemoglobin into the bloodstream [58]. ALT and GGTP activities in athletes can be used to assess the metabolic response to aerobic exercise [67]. In the studied groups of athletes, the measured GGTP and ALT activities increased after exercise and decreased after restitution. However, the corrected results did not show significant changes, showing that the changes were due to hemoconcentration. In another running test to exhaustion, an increase in GGTP activity was noted after exercise and a decrease to the baseline values 30 min after the end of the exercise. However, on the other hand, the corrected results showed that GGTP activity did not change [31].

In the study involving marathon runners, no increase in ALT activity was shown, respectively, 4 and 24 h after the end of the run [41]. In turn, after a 24-h ultramarathon, GGTP activity did not change, while ALT activity increased after the race and gradually decreased on days 2 and 9, remaining higher than before the race [45]. Participants in a 2-day ultramarathon did not show any change in GGTP activity. In contrast, ALT activity increased from day 2 to day 5 and decreased on day 7 but remained elevated compared to the baseline value [54]. The analysis of intensive and long-term runs is complemented by a study comparing the effect of long-distance runs on changes in blood parameters, which proved that the increase in ALT activity is greater the longer the run. In contrast, GGTP activity in the participants of this study increased only in people who ran 42 and 302 km and did not change significantly in people who ran 100 km [55]. However, after a 21-km run, no changes were found in ALT activity immediately after the run and at 2, 4, and 24 h after the run compared to the activity measured before the test [68].

Physical effort prevents osteoporosis and affects bone metabolism [57,58]. Calcium-phosphate metabolism and bone turnover in the studied groups of athletes and their dependence on physical effort were assessed in our studies. The assessments were evaluated based on the study of ALP activity and concentrations of phosphorus, total calcium, and ionized calcium. In both studied groups, a transient increase in ALP activity (measured) and phosphorus concentration and a transient decrease in total and ionized calcium concentration (before and after correction) were noted. A transient increase in ALP activity and phosphorus concentration, along with a persistently elevated total calcium concentration, was reported in marathon runners [41]. ALP activity in ultramarathon runners increased after a 24-h run but reached the baseline level on the second day [45]. A similar situation occurred in participants of a 60-min outdoor running test, which caused a transient increase in ALP activity [65]. In turn, a comparative study of long-distance running showed an increase in ALP activity after running only in marathon participants [55]. Another study showed that ALP activity did not change after an ultramarathon [69]. Running on a mechanical treadmill caused a transient increase in total calcium concentration, but correction of the results showed that after exercise, it was lower than before it, and after 30 min, it returned to the pre-test level [31]. Cycling a marathon distance led to a transient increase in ionized calcium concentration in cyclists [70], in contrast to an ultramarathon, which did not cause changes in the concentration of this parameter [67].

#### 4.2.3. The Changes in Concentration of Selected Metabolites as a Post-Effort Effect

Total and direct bilirubin levels are often elevated in athletes following vigorous exercise [56]. These studies only found changes in total and direct bilirubin levels in men. However, the results corrected for plasma volume change did not show any change from baseline. In a study of marathon runners, the total and direct bilirubin levels increased at 4 and 24 h after the race, but only direct bilirubin levels increased. In comparison, total bilirubin levels remained unchanged at 4 h [41]. Analysis of half-marathon runners showed increases in total and direct bilirubin levels immediately after the race, which continued for up to 24 h [68]. The effect of a 24-h ultramarathon was to increase the total and direct bilirubin levels immediately after the race and to decrease them on days 2 and 9 after the race [44]. A 2-day ultramarathon resulted in an increase in total and direct bilirubin concentrations on the second and third days after its completion. Between the fifth and seventh day, these values gradually returned to the baseline values [52]. A comparative analysis of long-distance runs demonstrated an increase in total and direct bilirubin concentrations in participants running 100 km and 302 km, without showing any changes in marathon runners who ran 42 km [53]. However, the presented results suggest that endurance exercise on a mechanical treadmill does not lead to changes in the concentrations of these analytes in the studied groups. The effect of physical exercise on kidney function in athletes from both groups was checked based on the concentrations of creatinine, urea, and uric acid. Creatinine concentrations in athletes are usually higher and depend on muscle mass, and urea and uric acid concentrations may increase as a result of physical exercise [59]. All participants in the current analysis had a transient increase in creatinine (both measured directly and corrected for plasma volume change), urea, and uric acid concentrations. Similar results were obtained in a study of cyclists who completed an ultramarathon—the concentrations of these parameters increased immediately after the race and decreased 24 h after its completion [67]. Other studies have shown that creatinine, urea, and UA levels rise and remain high 30 min after a treadmill endurance test to failure [31], up to 24 h after running a marathon [41], up to 24 h after a marathon bike ride [66], and up to 3 days after running a 2-day ultramarathon [52]. Increases in urea, creatinine, and UA levels immediately after exercise have also been noted in various long-distance races [53]. Another study of marathon runners demonstrated an increase in urea levels after the race and 24 h after its completion, and creatinine levels initially increased and then decreased to baseline values [69]. In a study of half-marathon runners, creatinine levels increased up to 24 h after the race [68]. A comparative study of half-marathon and marathon participants showed that creatinine levels increased in both groups due to running these distances [70].

Contrary to the results of Alis et al. (2016), where there was no post-effort increase in TG levels after the endurance test on a mechanical treadmill, there was no decrease in TG levels after the 30-min restitution period after the end of the test, and the total protein and cholesterol levels showed a transient post-effort decrease [27]. The results obtained in marathon runners indicated a transient increase in total protein levels. In turn, cholesterol and triglyceride levels in this group decreased significantly during this period [41]. In another study of ultramarathon runners, the total protein levels decreased immediately after the race [69]. A comparison of long-distance runs showed a decrease in total protein levels immediately after the 302 km race and an increase after the marathon [55]. In cyclists who completed a marathon, a decrease in the total protein concentration was observed only 24 h after the end of the race [70]. In turn, after a 24-h ultramarathon, the total protein and cholesterol concentrations decreased on day 2 and the triglycerides and LDL cholesterol decreased immediately after the race, while HDL cholesterol concentration remained unchanged [45]. In a study examining the effect of a 12-week aerobic training program, a decrease in total cholesterol, HDL cholesterol, and LDL cholesterol concentrations was observed [71].

The concentrations of the tested ions changed under the influence of physical effort. In the study of football players after the match, the concentration of potassium decreased, and the concentration of sodium did not show any significant changes [62]. In marathon participants, the concentration of sodium ions gradually decreased; the concentration of chloride ions was the lowest after four hours, the concentration of potassium ions decreased only after 24 h after the end of the race, and the concentration of magnesium decreased temporarily [41]. In cyclists who completed the marathon, similar relationships were observed—the concentration of sodium gradually reduced up to 24 h, the concentration of chlorides decreased the most immediately after the race, and the concentration of potassium remained unchanged [72]. In a study of marathon cyclists, there was a transient increase in potassium levels, whereas sodium, chloride, and magnesium levels did not change significantly within 24 h of completing the race [70].

Our research suggests that understanding the patient’s fitness level and the interval from the last training session before blood sampling can help prevent misinterpretation of laboratory test results, leading to incorrect diagnoses and subsequent negative consequences for both patients and the healthcare system. Extended testing exposes the patient to unnecessary stress and places a financial burden on the healthcare system. Therefore, it is crucial to include information on the time elapsed since the last training session before testing. For example, changes in liver enzyme activity after exercise may indicate liver disease [73,74,75], but the lack of changes in total and direct bilirubin levels suggests another cause. Similarly, elevated levels of total protein, albumin, urea, and uric acid may indicate metabolic diseases but may also be the body’s response to recent training [76].

It should be noted that these studies were conducted on regular sportsmen and sportswomen whose bodies are fully adapted to exercise. In more recreational athletes, changes in plasma volume and concentrations and activity of the tested parameters after exercise and after restitution may be different.

Every study has its limitations. In the case of our research, the low number of women participants may limit the findings in this group. Taking such uneven sex groups into account, the conclusions and generalizations of the results for clinical implications in the women’s group should be avoided. There could be a possible trend in the results visible, but further study on a larger women’s group is needed. Also, the hydration status and diet before testing may impact results. The participants were asked to eat a light breakfast and be properly hydrated. They assured us that they followed the recommendations, but they were not monitored. Therefore, body composition analysis should at least be included in the further study protocol. Another potential limitation of the test may be that blood was drawn either in a fasting or non-fasting state. However, current guidelines and recommendations allow for lipid profiling using blood samples taken from non-fasting individuals [77]. There is evidence that consuming a light meal in the morning does not significantly alter levels of total cholesterol, LDL-C, or HDL-C. However, triglyceride concentrations may increase by approximately 15% after food intake [78]. These minor and transient changes in lipid concentrations appear to be clinically insignificant.

## 5. Conclusions

In conclusion, the conducted studies may serve as a valuable tool for determining markers of post-exercise fatigue in young, trained men and women who train. In addition to CK, essential parameters in sports diagnostics may be changes in AST, ALT, and LDH activity, as well as concentrations of uric acid, potassium, total and ionized calcium. The conclusions obtained from the conducted studies may also contribute to better monitoring of training loads and regeneration strategies in people practicing other sports disciplines. The conducted analyses clearly show that laboratory tests performed in athletes may provide interpretation difficulties, and the reference ranges generally accepted in the healthy population do not apply to advanced amateur and professional athletes.

## Figures and Tables

**Table 1:** Participant characteristics.

Parameter	Men	Women
	*n* = 234	*n* = 62
Age (years)	19 (16–36)	21 (16–29)
Height (cm)	182 (179–185)	172 (165–176)
Weight (kg)	74.9 (69.7–80.0)	64.6 (61.0–68.7)
BMI (kg/m^2^)	22.6 (21.5–23.6)	22.3 (20.8–23.2)
VO_2_max	60.9 (58.4–64.4)	46.5 (42.5–50.4)
Length of training experience (years)	12 (10–14)	9 (6–10.5)
Weekly training volumes (h)	12 (10–14)	9 (6–12)

Data shows medians (interquartile ranges), except for age, which is presented as medians (minimum–maximum range). BMI—body mass index; VO_2_max—maximal aerobic capacity.

**Table 2:** Baseline, post-effort, and recovery blood morphology of the studied participants.

Variable	Time Point	Men *n* = 234	Women *n* = 62	p_MW_
RBC (10^9^/L)	baseline	5.1 ^aaa^ (4.8–5.3)	4.5 ^aaa^ (4.3–4.7)	<0.001
	post-effort	5.1 ^bbb^ (5.0–5.4)	4.6 ^bbb^ (4.4–4.9)	<0.001
	recovery	4.9 ^ccc^ (4.7–5.2)	4.4 ^ccc^ (4.2–4.6)	<0.001
HGB (mmol/L)	baseline	9.3 ^aaa^ (8.8–9.6)	8.0 ^aaa^ (7.6–8.3)	<0.001
	post-effort	9.5 ^bbb^ (9.0–9.8)	8.1 ^bbb^ (7.7–8.4)	<0.001
	recovery	9.1 ^ccc^ (8.5–9.5)	7.9 ^cc^ (7.3–8.2)	<0.001
HTC (L/L)	baseline	0.45 ^aaa^ (0.44–0.47)	0.40 ^aaa^ (0.38–0.41)	<0.001
	post-effort	0.47 ^bbb^ (0.45–0.49)	0.41 ^bbb^ (0.39–0.42)	<0.001
	recovery	0.45 ^ccc^ (0.43–0.46)	0.39 ^ccc^ (0.38–0.40)	<0.001
MCV (fL)	baseline	91 ^aaa^ (88–93)	88 (86–91)	<0.001
	post-effort	91 ^bbb^ (89–93)	88 ^bbb^ (86–91)	<0.001
	recovery	90 (88–93)	88 ^ccc^ (85–92)	0.001
MCH (fmol)	baseline	1.8 (1.8–1.9)	1.8 (1.7–1.8)	<0.001
	post-effort	1.8 (1.8–1.9)	1.8 (1.7–1.8)	<0.001
	recovery	1.8 (1.8–1.9)	1.8 ^c^ (1.8–1.9)	0.002
MCHC (mol/L)	baseline	20.3 ^a^ (19.6–20.8)	20.2 (19.1–20.5)	0.067
	post-effort	20.2 ^bbb^ (19.4–20.6)	20.2 (19.2–20.5)	0.234
	recovery	20.3 (19.5–20.8)	20.4 ^c^ (19.9–20.5)	0.316
RDW (%)	baseline	13.0 ^a^ (12.5–13.4)	13.5 (13.0–14.1)	<0.001
	post-effort	13.2 ^bbb^ (12.6–13.5)	13.6 (13.1–14.3)	<0.001
	recovery	13.0 (12.4–13.4)	13.5 (13.1–14.1)	<0.001
WBC (10^9^/L)	baseline	5.8 ^aaa^ (5.1–6.6)	6.6 ^aaa^ (5.9–7.5)	<0.001
	post-effort	9.0 ^bbb^ (7.9–10.3)	10.1 ^bbb^ (8.5–11.2)	0.002
	recovery	5.7 (5.0–6.5)	5.5 ^cc^ (4.9–6.5)	0.448
LYM (%)	baseline	36.6 ^aaa^ (30.6–41.5)	34.4 ^aaa^ (28.0–39.7)	0.077
	post-effort	42.2 ^bbb^ (36.6–47.9)	40.3 (35.1–46.0)	0.121
	recovery	37.2 (31.0–42.1)	38.9 ^ccc^ (33.4–45.0)	0.048
LYM (10^9^/L)	baseline	2.0 ^aaa^ (1.7–2.4)	2.2 ^aaa^ (1.9–2.6)	0.017
	post-effort	3.8 ^bbb^ (3.0–4.4)	4.0 ^bbb^ (3.4–4.4)	0.214
	recovery	2.0 ^c^ (1.6–2.3)	1.9 (1.7–2.4)	0.202
MON (%)	baseline	4.6 ^aaa^ (4.0–6.0)	3.8 ^aaa^ (3.1–4.5)	<0.001
	post-effort	5.2 ^bbb^ (4.5–6.5)	4.3 (3.7–5.1)	<0.001
	recovery	4.7 (3.9–6.1)	4.3 ^cc^ (3.7–4.9)	0.014
MON (10^9^/L)	baseline	0.2 ^aaa^ (0.2–0.3)	0.2 ^aaa^ (0.2–0.3)	0.005
	post-effort	0.4 ^bbb^ (0.3–0.6)	0.4 ^bbb^ (0.3–0.5)	<0.001
	recovery	0.2 (0.1–0.3)	0.2 (0.1–0.2)	0.063
GRA (%)	baseline	57.4 ^aaa^ (52.2–63.5)	61.3 ^aaa^ (56.0–68.9)	0.001
	post-effort	51.2 ^bbb^ (45.2–57.2)	55.5 (49.7–61.2)	0.001
	recovery	56.5 ^cc^ (51.4–62.2)	56.2 ^cc^ (49.2–62.2)	0.495
GRA (10^9^/L)	baseline	3.4 ^aaa^ (2.8–4.2)	4.3 ^aaa^ (3.5–5.0)	<0.001
	post-effort	4.6 ^bbb^ (3.8–5.5)	5.7 ^bbb^ (4.4–7.1)	<0.001
	recovery	3.3 (2.8–3.7)	3.2 ^ccc^ (2.6–3.9)	0.415
PLT (10^9^/L)	baseline	277 ^aaa^ (198–263)	261 ^aaa^ (227–298)	<0.001
	post-effort	292 ^bbb^ (247–319)	321 ^bbb^ (277–355)	0.001
	recovery	218 ^ccc^ (191–249)	241 ^cc^ (211–276)	<0.001
MPV (fL)	baseline	7.5 ^aa^ (6.9–7.9)	7.6 (7.3–8.2)	0.014
	post-effort	7.5 ^bbb^ (7.1–8.0)	7.8 ^bbb^ (7.2–8.4)	0.017
	recovery	7.4 ^c^ (6.9–7.8)	7.5 ^ccc^ (6.9–7.9)	0.353
PCT (10^−2^ L/L)	baseline	0.17 ^aaa^ (0.15–0.19)	0.20 ^aaa^ (0.18–0.22)	<0.001
	post-effort	0.21 ^bbb^ (0.19–0.24)	0.24 ^bbb^ (0.21–0.27)	<0.001
	recovery	0.16 ^ccc^ (0.14–0.18)	0.18 ^ccc^ (0.16–0.20)	<0.001
PDW (%)	baseline	16.0 ^aaa^ (15.1–16.8)	15.4 ^aa^ (14.8–15.9)	<0.001
	post-effort	16.0 ^b^ (15.2–17.0)	15.8 (15.1–16.7)	0.218
	recovery	15.8 (15.0–16.6)	15.3 ^c^ (14.8–:15.9)	0.012
ΔPV (%)	post-effort	−2.54 ^bbb^ (−3.99–0.709)	−1.92 ^bbb^ (−2.84–0.662)	0.027
	recovery	1.62 (−0.359–3.33)	1.86 (0.338–3.64)	0.411

Data are presented as medians (interquartile range). Significance levels of differences observed between analyzed time points (baseline vs. post-effort vs. recovery) were assessed using Friedman’s analysis of variance, followed by the post-hoc Dunn’s test with Bonferroni correction (Friedman’s ANOVA *p* values for each analysis were <0.001). Post-hoc *p* values: ^a^ *p* < 0.05, ^aa^ *p* < 0.01, and ^aaa^ *p* < 0.001 for baseline vs. post-effort; ^b^ *p* < 0.05, ^bbb^ *p* < 0.001 for post-effort vs. recovery; ^c^ *p* < 0.05, ^cc^ *p* < 0.01, and ^ccc^ *p* < 0.001 for recovery vs. baseline. p_MW_—*p*-values for differences observed between men vs. women assessed using the Mann–Whitney U-test; *n*—number of participants; RBC—red blood cells count; HB—hemoglobin; HTC—hematocrit; MCV—mean corpuscular volume; MCH—mean corpuscular hemoglobin; MCHC—mean corpuscular hemoglobin concentration; RDW—red blood cell distribution width; WBC—white blood cell count; LYM—lymphocytes; MON—monocytes; GRA—granulocytes; PLT—platelet count; MPV—mean platelet volume; PCT—plateletcrit; PDW—platelet distribution width; ΔPV—plasma volume loss.

**Table 3:** Serum ion concentrations in analyzed participants.

Variable	Time Point	Men *n* = 234	Women *n* = 62	*p*_MW_
Na^+^ (mmol/L)	baseline	137 ^aaa^ (136–138)	138 (137–139)	0.059
	post-effort	139 ^bbb^ (138–140)	139 ^bb^ (137–140)	0.166
	recovery	137 (136–138)	137 (136–139)	0.604
Corrected Na^+^ (mmol/L)	baseline	137 ^aaa^ (136–138)	138 ^aaa^ (137–139)	0.589
	post-effort	135 ^bbb^ (133–138)	136 ^bbb^ (134–138)	0.416
	recovery	140 ^ccc^ (136–143)	141 (137–142)	0.708
K^+^ (mmol/L)	baseline	4.02 ^aaa^ (3.89–4.21)	3.94 (3.83–4.12)	0.011
	post-effort	3.89 ^bbb^ (3.74–4.05)	3.89 (3.69–4.06)	0.839
	recovery	4.25 ^ccc^ (4.09–4.49)	3.95 (3.76–4.06)	<0.001
Corrected K^+^ (mmol/L)	baseline	4.02 ^aaa^ (3.89–4.21)	3.94 ^aa^ (3.83–4.12)	0.011
	post-effort	3.79 ^bbb^ (3.62–3.99)	3.83 ^bb^ (3.64–4.02)	0.496
	recovery	4.32 ^ccc^ (4.15–4.58)	3.98 (3.78–4.17)	<0.001
Cl^−^ (mmol/L)	baseline	98.9 ^aaa^ (97.9–100)	101 ^a^ (99.8–102)	<0.001
	post-effort	99.8 (98.6–101)	101 (99.9–102)	<0.001
	recovery	100 ^ccc^ (98.6–101)	101 (99.8–102)	<0.001
Corrected Cl^−^ (mmol/L)	baseline	98.9 ^aaa^ (97.9–100)	100.8 (99.8–102)	<0.001
	post-effort	97.2 ^bbb^ (95–99.5)	99.7 ^bbb^ (98.3–101)	<0.001
	recovery	102 ^ccc^ (98.6–104)	103 ^ccc^ (101–105)	0.010
tCa (mmol/L)	baseline	2.41 ^aaa^ (2.28–2.57)	2.31 (2.27–2.39)	<0.001
	post-effort	2.34 ^bbb^ (2.24–2.55)	2.29 ^bb^ (2.21–2.40)	0.003
	recovery	2.46 ^ccc^ (2.37–2.60)	2.33 (2.30–2.48)	<0.001
Corrected tCa (mmol/L)	baseline	2.41 ^aaa^ (2.28–2.57)	2.31 ^aa^ (2.27–2.39)	<0.001
	post-effort	2.29 ^bbb^ (2.19–2.51)	2.29 ^bbb^ (2.21–2.40)	0.003
	recovery	2.5 ^ccc^ (2.39–2.67)	2.38 ^c^ (2.30–2.48)	<0.001
iCa (mmol/L)	baseline	1.24 ^aaa^ (1.17–1.31)	1.19 ^aa^ (1.16–1.23)	<0.001
	post-effort	1.20 ^bbb^ (1.15–1.30)	1.17 ^bb^ (1.13–1.19)	<0.001
	recovery	1.25 ^ccc^ (1.21–1.31)	1.19 (1.17–1.23)	<0.001
Corrected iCa (mmol/L)	baseline	1.24 ^aaa^ (1.17–1.31)	1.19 ^aaa^ (1.16–1.23)	<0.001
	post-effort	1.17 ^bbb^ (1.11–1.26)	1.15 ^bbb^ (1.11–1.18)	0.029
	recovery	1.27 ^ccc^ (1.22–1.36)	1.22 (1.19–1.25)	<0.001

*p*_MW_—*p*-values for differences observed between men and women assessed using the Mann–Whitney U-test. Significance levels of differences observed between analyzed time points (baseline vs. post-effort vs. recovery) were assessed using Friedman’s analysis of variance, followed by post-hoc Dunn’s test with Bonferroni correction (Friedman’s ANOVA *p*-values for each analysis were <0.001). Post-hoc *p* values—^a^ *p* < 0.05, ^aa^ *p* < 0.01, and ^aaa^ *p* < 0.001 for baseline vs. post-effort; ^bb^ *p* < 0.01 and ^bbb^ *p* < 0.001 for post-effort vs. recovery; ^c^ *p* < 0.05 and ^ccc^ *p* < 0.001 for recovery vs. baseline. tCa—total Calcium; iCa—free unbound (ionized) calcium. Corrected values are after correction for hemoconcentration.

## Data Availability

The datasets generated and/or analyzed during the current study are available from the corresponding author on reasonable request.

## Supplementary Materials

The following supporting information can be downloaded at https://www.mdpi.com/article/10.3390/jcm14165703/s1: Table S1: The values of median, range, interquartile range, and 95% confidence intervals of analyzed variables; Min—minimum, Max—maximum, Q25, Q75—quartile values, CI—confidence intervals.

## Abbreviations

The following abbreviations are used in this manuscript:

ALP	alkaline phosphatase
ALT	alanine aminotransferase
AST	aspartate aminotransferase
ATP	adenosine triphosphate
BMI	body mass index
Ch-HDL	high-density lipoprotein cholesterol
Ch-LDL	low-density lipoprotein cholesterol
CK	creatine kinase
CRP	C-reactive protein
EDTA	ethylenediaminetetraacetic acid
GGTP	gamma-glutamyltransferase
GRA	granulocytes
HGB	hemoglobin
HTC	hematocrit
HDL	high-density lipoprotein
iCa	ionized calcium
LDH	lactate dehydrogenase
LDL	low-density lipoprotein
LYM	lymphocytes
MCH	mean corpuscular hemoglobin
MCHC	mean corpuscular hemoglobin concentration
MCV	mean corpuscular volume
MON	monocytes
MPV	mean platelet volume
PCT	plateletcrit
PDW	platelet distribution width
PLT	platelet count
RBC	red blood cells
RDW	red cell distribution width
TC	total cholesterol
tCa	total calcium
TG	triglycerides
TP	total protein
UA	uric acid
WBC	white blood cells
ΔPV	change in plasma volume
